# Clinical utility of blood neutrophil-lymphocyte ratio in Japanese COPD patients

**DOI:** 10.1186/s12890-018-0639-z

**Published:** 2018-05-02

**Authors:** Kaori Sakurai, Shotaro Chubachi, Hidehiro Irie, Akihiro Tsutsumi, Naofumi Kameyama, Takashi Kamatani, Hidefumi Koh, Takeshi Terashima, Hidetoshi Nakamura, Koichiro Asano, Tomoko Betsuyaku

**Affiliations:** 1 Division of Pulmonary Medicine, Department of Medicine, Keio University School of Medicine, 35 Shinanomachi, Shinjuku-ku, Tokyo, 160-8582 Japan; 2 Department of Medicine, Tachikawa Hospital, Tokyo, Japan; 3 Division of Pulmonary Medicine, Department of Internal Medicine, Tokyo Dental College, Chiba, Japan; 4 Department of Respiratory Medicine, Saitama Medical University, Saitama, Japan; 5 Division of Pulmonary Medicine, Department of Medicine, Tokai University School of Medicine, Kanagawa, Japan

**Keywords:** COPD, NLR, Acute exacerbation, Comorbidity

## Abstract

**Background:**

Neutrophil-to-lymphocyte ratio (NLR) is a biomarker of inflammation in chronic obstructive pulmonary disease (COPD) patients. But, a meaningful threshold and the longitudinal changes are unknown. We aimed to investigate the association between NLR and the clinical characteristics of COPD patients and to determine a meaningful threshold and the longitudinal changes for NLR.

**Methods:**

Keio University and its affiliate hospitals conducted an observational COPD cohort study over 3 years. We performed a blood examination and a pulmonary function test. Blood examination was completed at baseline and annually thereafter, at a time when the disease was stable. Two hundred seventy-four patients who had at least 3 blood examinations over 3 years were included.

**Results:**

Baseline NLR was correlated with baseline C-reactive protein (CRP) (*r* = 0.18, *p* = 0.003) and SAA (*r* = 0.34, *p* <  0.001). We defined an NLR score of 2.7 as the arbitrary cut-off value based on upper quartile points. COPD patients with NLR ≥ 2.7 were older (*p* = 0.037), had a lower BMI (*p* = 0.005) and a lower %FEV1 (*p* = 0.0003) compared to patients with NLR < 2.7. Receiver-operating-characteristic (ROC) curves showed the optimal cutoff for the baseline NLR in the predicting moderate/severe exacerbation to be 2.7, which was same as the upper quartile points. Follow-up analysis over 3 years revealed that the differences in the trends of NLR among the three groups based on the categories of exacerbations (moderate or severe, mild, no exacerbation) were significant (*p* = 0.006).

**Conclusions:**

NLR is associated with COPD severity and exacerbations. For predicting exacerbations, we estimated the threshold of NLR to be 2.7 at baseline.

**Trial registration:**

Clinical trial registered with the University Hospital Medication Information Network (UMIN000003470, April 10, 2010).

**Electronic supplementary material:**

The online version of this article (10.1186/s12890-018-0639-z) contains supplementary material, which is available to authorized users.

## Background

Chronic obstructive pulmonary disease (COPD) is common worldwide and is a major health-care concern [1]. COPD is characterized by low-grade chronic systemic inflammation [2], and several biomarkers such as C-reactive protein (CRP) [3, 4], IL-6 [4, 5] and surfactant protein D (SPD) [6] have been reported to be associated with increased risk of death in COPD patients. Although many biomarkers of systemic inflammation have recently been evaluated to identify some features of COPD [7, 8], excessive costs and technical factors prevent their clinical use.

Recently, blood neutrophil-to-lymphocyte ratio (NLR) has been shown to be a valuable predictor of inflammatory conditions and is used for risk stratification of different diseases such as acute coronary syndrome [9], pancreatitis [10], sepsis and infectious conditions [11]. This index is a rapid, easy and cost-effective method which is a calculated index derived from a routine complete blood count test in clinical practice [12]. NLR has been reported to be higher in exacerbated COPD patients than in stable patients [13] and is associated with severity of COPD [14, 15]. As with other inflammatory markers, NLR has been reported to vary with time [16, 17]. However, annual time-series data of individual NLR, its distribution and a meaningful threshold in COPD remain to be investigated.

Comorbidities and extrapulmonary manifestations have important contributions to disease expression, disease burden, and survival in COPD patients [18]. Several comorbidities such as heart disease, hypertension, and diabetes [19] are also associated with systemic inflammation. However, the association between these comorbidities and NLR in COPD patients has not been elucidated.

We have been conducting a multicenter, observational cohort study, called the Keio COPD Comorbidity Research (K-CCR), to comprehensively examine the comorbidities of COPD in Japan [20–22].

The specific aims of this study are to determine (a) whether NLR is a biomarker that can reflect the severity of airflow limitation and certain other characteristics of COPD patients, (b) if it can predict future exacerbations and (c) if it is associated with any comorbidities. We thus monitored NLR and other known blood biomarkers in Japanese COPD patients over 3 years to evaluate its stability as a biomarker in patients at steady state and investigated its association with a variety of clinical aspects of COPD.

## Methods

### Study population

**Fig. 1:**
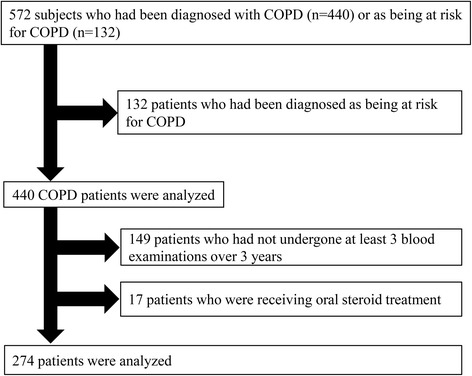
Process of patient selection in this study. Data from only those COPD patients with spirometrically confirmed COPD (FEV1/forced vital capacity (FVC) < 0.7) who had undergone at least 3 blood examinations over 3 years and were not receiving any oral steroid treatments (*n* = 274) were analyzed. COPD, chronic obstructive pulmonary disease

The overall design of the K-CCR has been previously published [20, 22, 23]. In brief, this study was a 3-year, prospective observational study that enrolled 572 men and women aged 40–91 years who had been diagnosed with COPD (*n* = 440) or as being at risk for COPD (*n* = 132) by pulmonary physicians from April 2010 to December 2012 [20, 22, 23]. For the purpose of this study, only data from the patients with spirometrically confirmed COPD (FEV1/forced vital capacity (FVC) <  0.7) who had undergone at least 3 blood examinations over 3 years and were not receiving any oral steroid treatments (*n* = 274) were selected and analyzed (Fig. 1). All patients were clinically stable and had no exacerbations and no acute infections for at least 1 month prior to recruitment [20, 22, 23]. The ethics committees of the Keio University and its affiliated hospitals approved the study protocol. Each patient provided written, informed consent to analyze and present their data. All aspects of the study conformed to the principles of the Declaration of Helsinki adopted by the 59th WMA General Assembly, Seoul, Republic of Korea, October 2008.

### Assessment of clinical parameters

Complete medical and smoking histories, as well as information regarding the current pharmacologic treatments, were obtained on enrolment and annually thereafter [20, 22]. All patients were assessed by spirometry and chest computed tomography (CT) imaging. On the CT images, the extent of emphysema was quantified as the ratio of low attenuation area (LAA %) and the percentage of airway wall area (WA %) using a custom-made software (AZE Ltd., Tokyo, Japan) [22, 23]. Independent investigators judged the number and severity of exacerbations based on the reviews of physicians’ medical records, as previously reported [22, 24]. Mild COPD exacerbation was defined as worsening of symptoms that were self-managed (by measures such as an increase in salbutamol use) and resolved without systemic corticosteroids or antibiotics [24]. Moderate COPD exacerbation was defined by the requirement for treatment with systemic corticosteroids and/or antibiotics [24]. Severe COPD exacerbation was defined as one that required hospitalization, including an emergency admission for 24 h [24]. Comorbid diagnoses were established by clinical history and examination findings based on a review of available medical records [20, 21, 23, 24].

### Evaluation of neutrophil-to-lymphocyte ratio (NLR) and other inflammatory markers

Blood samples were collected at baseline and annually thereafter, at a time when the disease was stable. The total numbers of white blood cells (WBCs), neutrophils, and lymphocytes were measured at each participating center. NLR was defined as the absolute neutrophil count divided by the absolute lymphocyte count [9]. Serum C-reactive protein (CRP) levels were measured using a Hitachi Ltd. LABOSPECT 008 devise. Serum amyloid A (SAA) is an acute-phase protein similar to CRP, and its levels were evaluated by latex agglutination immunoassay. The annual rates of change in NLR were measured over 3 years. Excel (Microsoft Inc., Redmond, WA, USA) was used to calculate the derived slope between each of the above measures and the date of data collection [22, 25].

### Questionnaires on health-related quality of life

All patients were clinically stable and had no exacerbations for at least 1 month before study enrollment and on the day of the annual examination [22]. The Japanese version of COPD assessment test (CAT) [26, 27] and the St. George’s Respiratory Questionnaire (SGRQ) in Japanese were used for the assessment of COPD-specific health status [28–30]. All of the questionnaires were completed by the patients themselves at home [20].

### Statistical analysis

Data are presented as the mean ± standard deviation (SD) or as median (interquartile range (IQR)). Data were compared between two groups using the student *t*-test, Mann-Whitney U test, and χ^2^ test. Data were compared among three or more groups using Kruskal-Wallis test and Steel-Dwass test. Correlations between continuous variables were evaluated using the Pearson’s correlation coefficient. Univariate and multivariate logistic regression analyses were performed in order to assess the effects of factors on increasing NLR. Receiver operating characteristics (ROC) curves were constructed to assess the areas under the curves (AUCs). We investigated the optimal cutoff value by maximizing the Youden index. The data of NLR over 3 years were only used in the analysis of the annual rates of change in NLR, and in the comparison of the average of NLR among the three groups over 3 years. Differences in rates of change over time and in NLR among the 3 groups classified based on the severity of exacerbation were estimated using mixed-effects modeling with Bonferroni correction [22]. For all tests, two-sided *p* values < 0.05 were considered significant. Data were analyzed using the JMP 13 software (SAS Institute, Cary, NC, USA). A mixed-effect model was applied using SPSS 23 (IBM Corporation, Armonk, NY, USA).

## Results

### Characteristics of the study population

Table 1 shows the baseline clinical characteristics of the study patients. The mean age of the 274 COPD patients was 72.2 ± 7.9 years, and 9.6% of them were current smokers. At baseline, 23.0%, 48.9%, 22.3%, and 5.8% were diagnosed as COPD grade 1, 2, 3, and 4, respectively based on the Global Initiative for Chronic Obstructive Lung Disease (GOLD) classification [31]. Baseline NLR significantly increased with the severity of COPD grade (1.9 (1.4–2.4) vs. 2.1 (1.5–2.6) vs. 2.3 (2.0–3.0) vs. 2.7 (1.9–5.3), *p* = 0.002) (Table 2).

**Table 1:** Baseline clinical characteristics of the study patients

	COPD
Number	274
Gender, female, *n* (%)	17 (6.2)
Age, years	72.2 ± 7.9
Smoking Index, pack-years	55.2 ± 29.7
Current smokers, *n* (%)	26 (9.6)
BMI, kg/m^2^	22.9 ± 3.1
FEV_1_/FVC, %	52.4 ± 12.3
% FEV_1_, %	63.3 ± 21.1
GOLD grade 1/2/3/4, *n* (%)	63/134/61/16 (23.0/48.9/22.3/5.8)
Baseline CAT score	12.2 ± 8.0
LAMA, *n* (%)	170 (62.0)
LABA, *n* (%)	136 (49.6)
ICS, *n* (%)	93 (33.9)
Dose of ICS^a^, μg/day, median	500
WBC, *n*	6208 ± 1566
Neutrophil, %	60.3 ± 8.9
Neutrophil, *n*	3797 ± 1320
Lymphocyte, %	29.2 ± 8.2
Lymphocyte, *n*	1770 ± 553
NLR	2.1(1.6–2.7)
Eosinophil, %	3.4 ± 2.6
Eosinophil, *n*	204 ± 150

Data are shown as mean ± SD and median (interquartile range)

*COPD* chronic obstructive pulmonary disease, *BMI* body mass index, *FEV*_*1*_ forced expiratory volume in one second, *FVC* forced vital capacity, *% FEV*_*1*_ ratio of predicted FEV_1_, *GOLD* Global Initiative for Chronic Obstructive Lung Disease, *CAT* COPD assessment test, *LAMA* long-acting muscarinic antagonist, *LABA* long-acting β_2_ agonist, *ICS* inhaled corticosteroids, *WBC* white blood cell, *NLR* Neutrophil-to-Lymphocyte ratio

^a^Dose of inhaled corticosteroid is shown as fluticasone propionate equivalent

**Table 2:** Comparison of the baseline NLR stratified by the GOLD COPD grade

GOLD COPD grade	NLR
1	1.9 (1.4–2.4)
2	2.1 (1.5–2.6)
3	2.3 (2.0–3.0)*
4	2.7 (1.9–5.3)

Data are shown as median (interquartile range). *P*-values among the four groups; *p* = 0.002, *p = 0.008 vs. grade 1

*GOLD* Global Initiative for Chronic Obstructive Lung Disease, *COPD* chronic obstructive pulmonary disease, *NLR* Neutrophil-to-Lymphocyte ratio

### Comparison of patient characteristics based on baseline NLR

NLR data showed a non-normal distribution. The median baseline NLR was 2.1(1.6–2.7). Baseline characteristics of patients according to the baseline NLR are shown in Table 3. The cut-off value of NLR was arbitrarily defined based on the upper quartile points as 2.7. COPD patients with NLR ≥ 2.7 (*n* = 66) were older (74.0 ± 7.1 years vs. 71.6 ± 8.1 years, *p* = 0.037), had a lower BMI (22.0 ± 3.4 vs. 23.2 ± 2.9, *p* = 0.005) and a lower % FEV_1_ (55.8 ± 21.3% vs. 66.5 ± 20.4%, *p* = 0.0003) compared to patients with NLR < 2.7 (*n* = 199). Baseline NLR did not differ between current smokers and past smokers (2.12 ± 0.24 vs. 2.38 ± 0.08, *p* = 0.30). On CT images, LAA% of the patients with NLR ≥ 2.7 tended to be higher than that of the others (18.4 (6.4–28.7) % vs. 11.3 (5.0–23.3) %, *p* = 0.093), while WA% did not differ between the two groups (54.9 (46.4–59.8) % vs. 52.4 (47.4–57.7) %, *p* = 0.416). The COPD patients with NLR ≥ 2.7 exhibited a significantly higher total CAT score (13.9 ± 7.7 vs. 11.5 ± 8.0, *p* = 0.039) and SGRQ total score (36.2 ± 19.1 vs. 24.8 ± 18.0, *p* < 0.001) compared to the others.

**Table 3:** Patient characteristics based on baseline NLR

	NLR < 2.7	NLR ≥ 2.7	*p*-value
Number	199	66	
Gender, female, *n* (%)	14 (7.0)	3 (4.6)	0.474
Age, years	71.6 ± 8.1	74.0 ± 7.1	0.037
Smoking Index, pack-years	53.7 ± 28.8	59.9 ± 33.8	0.159
Current smokers, *n* (%)	21 (10.7)	4 (6.1)	0.270
BMI, kg/m^2^	23.2 ± 2.9	22.0 ± 3.4	0.005
FEV_1_/FVC, (%)	53.8 ± 11.8	48.9 ± 13.3	0.005
% FEV_1_, (%)	66.5 ± 20.4	55.8 ± 21.3	0.0003
LAA%, (%)	11.3 (5.0–23.3)	18.4 (6.4–28.7)	0.093
WA%, (%)	52.4 (47.4–57.7)	54.9 (46.4–59.8)	0.416
Baseline CAT score	11.5 ± 8.0	13.9 ± 7.7	0.039
SGRQ total score	24.8 ± 18.0	36.2 ± 19.1	< 0.001
LAMA, *n* (%)	120 (60.3)	44 (66.7)	0.356
LABA, *n* (%)	94 (47.2)	40 (60.6)	0.060
ICS, *n* (%)	63 (31.7)	28 (42.4)	0.111
Dose of ICS^a^, μg/day, median	500	500	0.980
Eosinophil, (%)	3.6 ± 2.6	2.9 ± 2.4	0.080
Eosinophil, *n*	209 ± 149	188 ± 153	0.318

Data are shown as mean ± SD and median (interquartile range)

*COPD* chronic obstructive pulmonary disease, *NLR* Neutrophil-to-Lymphocyte ratio, *BMI* body mass index, *FEV*_*1*_ forced expiratory volume in one second, *FVC* forced vital capacity, *% FEV*_*1*_ ratio of predicted FEV_1_, *LAA%* ratio of low attenuation area, *WA%* ratio of airway wall area, *CAT* COPD assessment test, *SGRQ* St. George’s Respiratory Questionnaire, *LAMA* long-acting muscarinic antagonist, *LABA* long-acting β_2_ agonist, *ICS* inhaled corticosteroids, *CRP* C-reactive protein, *SAA* serum amyloid A

^a^Dose of inhaled corticosteroid is shown as fluticasone propionate equivalent

### Relationship between NLR and other inflammatory markers

Baseline NLR showed a weak but significant correlation with baseline CRP (*r* = 0.18, *p* = 0.003) and SAA (*r* = 0.34, *p* < 0.001). Similarly, baseline absolute neutrophil count also correlated significantly with baseline CRP (*r* = 0.24, *p* < 0.001) and baseline SAA (*r* = 0.31, *p* < 0.001), but the baseline absolute lymphocyte count did not show correlation with the other inflammatory markers. The absolute neutrophil count showed no correlation with the absolute lymphocyte count (*r* = 0.08, *p* = 0.214). These results imply that the absolute neutrophil count has a bigger impact on the value of NLR as systemic inflammation.

### Determinant factors of NLR

Univariate logistic regression analysis showed that high age, % FEV_1_ < 50%, low BMI (BMI < 18.5), high CRP and high SAA were all significantly associated with NLR ≥ 2.7 (Additional file 1: Table S1). We then performed a multivariate logistic regression analysis including the associating factors that reached significance in the univariate analysis. Because of the significant correlations between SAA and CRP (*r* = 0.84, *p* < 0.001), SAA was excluded from the multivariate analysis. % FEV_1_ < 50%, low BMI, and high CRP were found to be independent determinant factors of NLR ≥ 2.7 (Table 4). These results imply that NLR is multifactorial, and is independently associated with severe airflow limitation, advanced age, and low BMI. Also, it is reflective of other systemic inflammation markers.

**Table 4:** Predictors of high NLR (NLR ≥ 2.7) by multivariate logistic regression analysis

	Odds ratio (95% CI)	*P*-value
Age	1.04 (0.99–1.08)	0.063
BMI < 18.5	2.95 (1.22–7.09)	0.016
% FEV_1_ < 50%	2.35 (1.25–4.41)	0.008
CRP	1.89 (1.08–3.28)	0.008

*NLR* Neutrophil-to-Lymphocyte ratio, *BMI* body mass index, *FEV*_*1*_ forced expiratory volume in one second, *% FEV*_*1*_ ratio of predicted FEV_1_, *CRP* C-reactive protein

### Comparison of comorbidity prevalence according to the baseline NLR

Prevalence of comorbidity in the study patients according to the baseline NLR is shown in Table 5. The prevalence of arteriosclerosis obliterans, cancer and cataract were tended to be higher in the NLR ≥ 2.7 group than in the NLR < 2.7 group (arteriosclerosis obliterans; 0.5% vs. 3.0%, *P* = 0.096, caner; 18.4% vs. 28.7%, *p* = 0.072, cataract; 38.7% vs. 53.3%, *p* = 0.081). There were no statistical differences in the prevalence of cardiovascular disease, hypertension, diabetes. These results imply that NLR is not influenced by comorbidities in COPD patients.

**Table 5:** Prevalence of comorbidity in the study patients according to the baseline NLR

*n* (%)	NLR < 2.7	NLR ≥ 2.7	p-value
Benign prostatic hyperplasia	30 (15.3)	10 (15.2)	0.976
Tuberculosis	17 (8.7)	5 (7.6)	0.781
Arteriosclerosis obliterans	1 (0.5)	2 (3.0)	0.096
Aortic aneurysm	7 (3.6)	2 (3.0)	0.835
Arrhythmia	23 (11.7)	6 (9.1)	0.554
Heart failure	13 (6.6)	2 (3.0)	0.276
Coronary artery disease	23 (11.7)	8 (12.1)	0.933
Peptic ulcer disease	13 (6.6)	4 (6.1)	0.870
Dyslipidemia	35 (17.9)	10 (15.2)	0.614
Diabetes mellitus	30 (15.3)	8 (12.1)	0.525
Hypertension	71 (36.2)	23 (34.9)	0.840
Interstitial pneumonia	38 (26.8)	12 (35.3)	0.322
Pneumothorax	10 (5.1)	3 (4.6)	0.857
Hyperuricemia	16 (8.2)	6 (9.1)	0.814
Cerebral infarction	11 (5.6)	5 (7.6)	0.565
Asthma (Asthma COPD overlap)	38 (19.5)	16 (24.2)	0.410
Depression	15 (7.7)	9 (14.1)	0.124
Cancer	36 (18.4)	19 (28.7)	0.072
Cataract	58 (38.7)	24 (53.3)	0.081

*NLR* Neutrophil-to-Lymphocyte ratio, *COPD* chronic obstructive pulmonary disease

### Longitudinal changes in NLR over 3 years

**Fig. 2:**
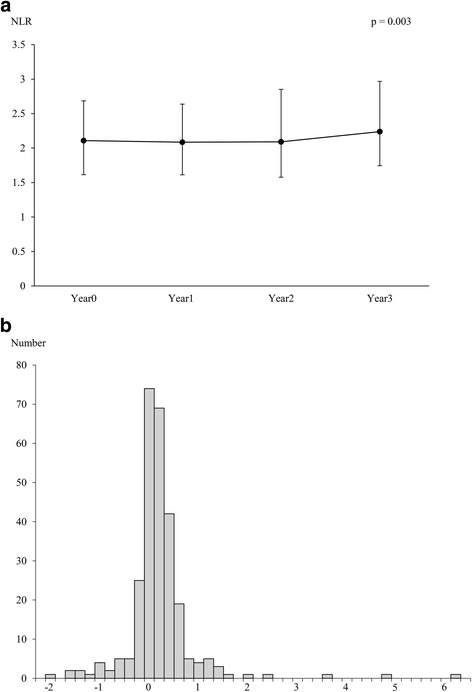
Longitudinal changes in NLR. **a** Time-dependent changes in NLR. **b** The distribution of the annual rate of change in NLR (ΔNLR score/year) over 3 years. NLR, Neutrophil-to-Lymphocyte ratio; ΔNLR, the rate of change in NLR

Overall, time-dependent changes in NLR showed a slight increase over 3 years (*p* = 0.003) (Fig. 2a). The distribution of the annual rate of change in NLR (ΔNLR score/year) over 3 years is shown in Fig. 2b. The median ΔNLR score/year was 0.05 (− 0.15–0.25).

### Relationship between NLR and COPD exacerbation over 3 years

**Fig. 3:**
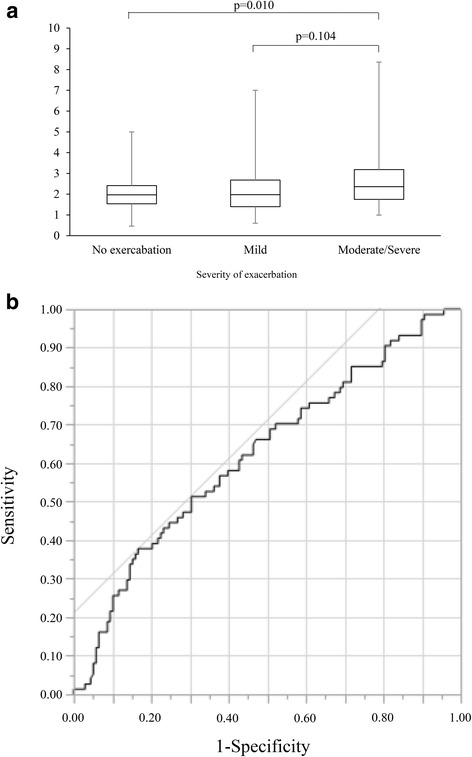
Relationship between NLR and COPD exacerbation. **a** Comparison of median baseline NLR based on the severity of exacerbation over 3 years. **b** ROC curves of median baseline NLR in the predicting moderate or severe exacerbation. Data were compared among three groups using Steel-Dwass test. NLR, Neutrophil-to-Lymphocyte ratio

**Fig. 4:**
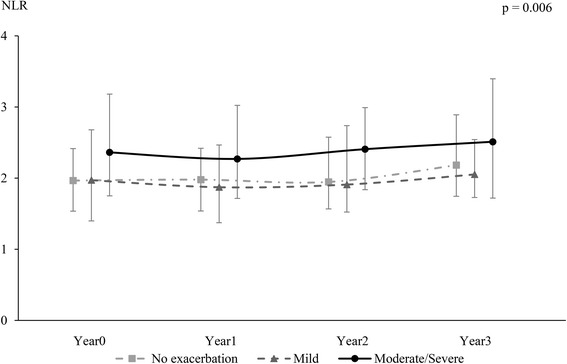
Trend in NLR changes over 3 years. The trend of NLR changes in the none, mild and moderate/severe exacerbations groups was significantly different (*p* = 0.006). Moderate/severe exacerbation vs exacerbation-free, *p* = 0.014; Moderate/severe exacerbation vs mild exacerbation, *p* = 0.022. NLR, Neutrophil-to-Lymphocyte ratio

Based on the degree of exacerbations defined in our previous paper [24], the COPD subjects were classified into 3 groups: without exacerbations (*n* = 86), only mild exacerbations (*n* = 52), and moderate or severe exacerbations (*n* = 74). Among the 3 groups, the median baseline NLR was significantly higher in the moderate/severe exacerbation group compared to the exacerbation-free group but it was not significantly different when compared to the mild exacerbation group (2.4 (1.8–3.2) vs. 2.0 (1.5–2.4) and 2.0(1.4–2.7), *p* = 0.010 and 0.104, respectively) (Fig. 3a). Univariate logistic regression analysis showed that the baseline NLR ≥ 2.7 (OR; 2.89, *p* = 0.001), Age (OR; 1.05, p = 0.01), %FEV_1_ < 50 (OR; 3.23, *p* = 0.0002) and LAA% (OR; 1.05, *p* = 0.0004) were significant predictors of moderate/severe exacerbation (Additional file 1: Table S2). Multivariate logistic regression analysis was performed using risk factors that reached significance on univariate analyses. Because of the significant negative correlations between %FEV_1_ and LAA% (*r* = − 0.47, *p* < 0.0001), LAA% were excluded from multivariate analysis. The baseline NLR ≥ 2.7 was an independent risk factor for moderate exacerbations over 3 years (OR; 2.22, *p* = 0.025) (Additional file 1: Table S3). ROC curves showed that the optimal cutoff for the baseline NLR for predicting moderate/severe exacerbations was 2.7 (AUC 62.3, sensitivity 37.8%, specificity 83.3%) (Fig. 3b). Follow-up analysis over 3 years revealed that there was a significant difference in the average value of NLR among the three groups (*p* = 0.006), but the trends of NLR were comparable among the three groups over 3 years (*p* = 0.476) (Fig. 4).

### Comparison of receiver-operator curves for NLR, neutrophil and lymphocyte counts, and other biomarkers to stratify the severity of airflow limitation and predict future exacerbations

The area under ROC for NLR to predict future exacerbation was 63.4% (95% CI 55.0–71.7) and the baseline low % FEV_1_ (%FEV_1_ < 50%) was 64.2% (95% CI 56.9–71.6), and both seemed to be better than the baseline absolute neutrophil and lymphocyte counts alone, CRP and SAA, implying that NLR was a superior predictor of the severity of airflow limitations and future exacerbations compared to the neutrophil and lymphocyte counts alone (Table 6).

**Table 6:** Comparison of the areas under ROC for NLR and other biomarkers to predict future exacerbations and low % FEV_1_ (% FEV_1_ < 50%)

Exacerbation	AUC (95% CI)
NLR	63.4 (55.0–71.7)
Neutrophil count	55.2 (46.3–64.2)
Lymphocyte count	59.2 (50.8–67.6)
Eosinophil count	51.2 (41.9–60.6)
CRP	54.3 (45.2–63.4)
SAA	59.4 (50.5–68.3)
% FEV_1_ < 50%	AUC (95% CI)
NLR	64.2 (56.9–71.6)
Neutrophil count	55.3 (47.3–63.2)
lymphocyte count	59.8 (52.3–67.3)
Eosinophil count	51.5 (43.4–59.6)
CRP	55.5 (47.7–63.5)
SAA	57.7 (49.8–65.6)

*NLR* Neutrophil-to-Lymphocyte ratio, *CRP* C-reactive protein, *SAA* serum amyloid A

## Discussion

### Mechanism of the relationship between NLR and COPD severity

Consistent with a previous report [14], in this study, we found that NLR was associated with COPD severity and exacerbations. Several studies have reported the appropriate thresholds to predict the natural history of systemic diseases other than COPD. In this study, we revealed the appropriate cut-off value of NLR as 2.7 to predict COPD severity and future exacerbations [32, 33]. The mechanisms underlying these relationships are unknown, but there could be several explanations. Firstly, it is well known that, even after smoking cessation, inflammation in the lungs continue, especially in patients with advanced COPD [34], suggesting that the persistent inflammatory response in the lungs could lead to neutrophil recruitment and activation [35]. When activated, neutrophils release a variety of serine- and metalloproteinases, which contribute to the development and progression of emphysema [36]. It has been shown that sputum neutrophilia is increased in advanced COPD and is associated with severity of airflow limitation [37]. Blood neutrophilia is a hallmark of current smokers [38] and is also a predictor of mortality in COPD patients [4]. Secondly, a relationship between bacterial colonization and exacerbations is increasingly recognized [39]. Thus, in some patients with COPD, the disturbed flora may continue to activate the innate immune responses, perpetuating lung inflammation and blood neutrophilia. We would like to emphasize that most of the participants in this study were not current smokers. Thirdly, blood lymphopenia is associated with age [40] and poor nutritional status [41], which could also be applicable to a subset of COPD patients. Taken together, NLR along with age and nutritional status could be reflective of the systemic inflammatory condition. Weak correlations between NLR and other biomarkers might be caused by multifactorial determinants of NLR.

### NLR and COPD comorbidities

It should be noted that this is the first study to assess the relationships between NLR and COPD comorbidities. Chronic systemic inflammation which is a characteristic of most comorbidities is believed to be a key factor that links COPD with its comorbidities [42]. In the ECLIPSE cohort, most COPD patients with heart disease had elevated IL-6, IL-8, and fibrinogen, while those with hypertension had elevated fibrinogen and those with diabetes had elevated CRP [19]. In our study, these comorbidities were not associated with NLR. It would, therefore, be unreliable to predict the presence of comorbidities by the serum inflammatory markers in COPD patients.

### Clinical usefulness of NLR

NLR is a new addition to the list of inflammatory markers and has got special attention in the recent years. The value for NLR is obtained by dividing the absolute neutrophil count by the absolute lymphocyte count in peripheral blood samples. Moreover, this test is quite inexpensive, usually not requiring a proper setup and can be used as a screening and drug monitoring tool in population on a large scale [43].

Strategies to prevent exacerbations include targeting risk factors, addressing comorbid conditions, and giving bronchodilator therapies which include long-acting β2-agonists (LABAs) or long-acting muscarinic antagonists (LAMAs) used alone or in combination with each other or with an inhaled corticosteroid [ICS]) [44, 45]. A number of studies have shown that the higher the blood eosinophil count, higher is the risk of exacerbation [46] and greater is the exacerbation reduction response to inhaled corticosteroids [47, 48]. In this study, although NLR was a predictor of exacerbations, blood eosinophilia was not a risk factor. This may be because the prescription rate of ICS was high in our populations than in the other cohort studies. One previous report has shown that the count and percent of blood eosinophils were not associated with the exacerbation frequency in COPD patients after carefully excluding the Asthma–COPD overlap syndrome (ACOS) patients [49]. ICS may be insufficient to prevent exacerbations in COPD patients with a high NLR, and a new prophylactic therapy should be considered. NLR may be a simple and inexpensive biomarker for detecting a neutrophilic endotype, and this endotype may be indicative of the new pharmacological strategy for treating the exacerbation. In addition to the usual care, azithromycin and roflumilast are likely candidates for prophylactic therapy in these patients. Azithromycin and roflumilast treated patients had lower sputum proline-glycine-proline (PGP) levels and showed a reduction in other markers of neutrophilic inflammation [50, 51].

### Limitations of this study

Our study had several limitations. First, although NLR was associated with exacerbations over 3 years, outcomes such as hospital admissions, emergency room visits, and mortality remain to be evaluated. Sørensen et al. reported that NLR was associated with higher mortality in moderate to very severe COPD patients [52], but it is unknown whether this result can be applied to milder COPD patients in our study. Second, blood NLR may not accurately reflect the lung inflammation of COPD patients. Low sensitivity of NLR to predict future exacerbations and airflow limitation severity may be caused by this reason. It would be preferable if we could compare blood NLR and biomarkers or cell fractionation of bronchoalveolar lavage and sputum. Third, COPD patients of the KCCR cohort study had few women (6.2%) and were older compared to subjects of other clinical studies in Western countries. Therefore, the results may not apply to women and younger COPD patients.

## Conclusions

The present study demonstrates that NLR is associated with COPD severity and exacerbations, but not with systemic comorbidity in COPD patients. For predicting exacerbations, we estimated the threshold of NLR to be 2.7 at baseline. This information could be useful in the phenotyping of COPD patients, and their careful monitoring in clinical settings. Appropriate interventions for these patients should be considered in future.

## Additional file

Additional file 1: **Table S1.** Predictors of high NLR (NLR ≥ 2.7) by univariate logistic regression analysis. **Table S2.** Predictors of moderate or severe exacerbation by univariate logistic regression analysis. **Table S3.** Predictors of moderate or severe exacerbation by multivariate logistic regression analysis. (DOCX 19 kb)

## Abbreviations

%FEV_1_: Ratio of predicted FEV_1_
BMI: Body mass index
CAT: COPD assessment test
COPD: Chronic obstructive pulmonary disease
CRP: C-reactive protein
FEV_1_: Forced expiratory volume in one second
FVC: Forced vital capacity
GERD: Gastroesophageal reflux disease
GOLD: Global Initiative for Chronic Obstructive Lung Disease
HAD-A: Seven items for anxiety of hospital anxiety and depression
HAD-D: Seven items for depression of hospital anxiety and depression
ICS: Inhaled corticosteroids
LAA%: Ratio of low attenuation area
LABA: Long-acting β_2_ agonist
LAMA: Long-acting muscarinic antagonist
NLR: Neutrophil-to-Lymphocyte ratio
SAA: Serum amyloid A
SGRQ: St. George’s Respiratory Questionnaire
WA%: Ratio of airway wall area
ΔNLR: Rate of change in NLR
