# Supplementary material for: Clinical utility of blood neutrophil-lymphocyte ratio in Japanese COPD patients
Source: BMC Pulm Med. 2018 May 2;18:65. doi: 10.1186/s12890-018-0639-z (PMC5932787; doi:10.1186/s12890-018-0639-z)
Supplement: Supplementary file 1 — Table S1. Predictors of high NLR (NLR ≥ 2.7) by univariate logistic regression analysis. Table S2. Predictors of moderate or severe exacerbation by univariate logistic regression analysis. Table S3. Predictors of moderate or severe exacerbation by multivariate logistic regression analysis. (DOCX 19 kb) [file 12890_2018_639_MOESM1_ESM.docx]

**Table S1** Predictors of high NLR (NLR ≥ 2.7) by univariate logistic regression analysis.

|  | Odds ratio (95%CI) | p-value |
| --- | --- | --- |
| Gender, female | 0.63 (0.14-2.01) | 0.459 |
| Age | 1.04 (1.00-1.08) | 0.034 |
| Smoking Index, pack-years | 1.01 (0.99-1.02) | 0.165 |
| Current smokers | 0.54 (0.15-1.49) | 0.249 |
| BMI < 18.5 | 2.64 (1.14-6.15) | 0.024 |
| % FEV_1_ | 0.97 (0.96-0.99) | < 0.001 |
| % FEV_1_ < 50% | 2.67 (1.47-4.84) | < 0.001 |
| LAA% | 1.02 (0.99-1.05) | 0.054 |
| WA% | 1.02 (0.97-1.06) | 0.486 |
| Baseline CAT score | 1.04 (1.00-1.07) | 0.042 |
| LAMA | 1.32 (0.74-2.39) | 0.353 |
| LABA | 1.72 (0.98-3.06) | 0.059 |
| ICS | 1.59 (0.89-2.82) | 0.114 |
| CRP | 2.01 (1.28-3.92) | < 0.001 |
| SAA | 1.02 (1.01-1.06) | < 0.001 |

NLR, Neutrophil-to-Lymphocyte ratio; BMI, body mass index; FEV_1_, forced expiratory volume in one second; %FEV_1_, ratio of predicted FEV_1_; LAA%, ratio of low attenuation area; WA%, ratio of airway wall area; LAMA, long-acting muscarinic antagonist; LABA, long-acting β_2_ agonist; ICS, inhaled corticosteroids; GERD, gastroesophageal reflux disease; HAD-A, seven items for anxiety of hospital anxiety and depression; HAD-D, seven items for depression of hospital anxiety and depression; CRP, C-reactive protein; SAA, serum amyloid A

**Table S2** Predictors of moderate or severe exacerbation by univariate logistic regression analysis.

|  | Odds ratio (95%CI) | p-value |
| --- | --- | --- |
| Gender, female | 0.35 (0.05-1.39) | 0.146 |
| Age | 1.05 (1.01-1.10) | 0.010 |
| Smoking Index, pack-years | 1.01 (1.00-1.02) | 0.014 |
| Current smokers | 0.52 (0.16-1.37) | 0.192 |
| BMI < 18.5 | 1.59 (0.61-4.02) | 0.335 |
| % FEV_1_ < 50% | 3.23 (1.74-6.09) | 0.0002 |
| LAA% | 1.05 (1.02-1.08) | 0.0004 |
| WA% | 1.02 (0.98-1.06) | 0.292 |
| CRP | 1.19 (0.87-1.72) | 0.268 |
| SAA | 1.00 (1.00-1.01) | 0.252 |
| NLR ≥2.7 | 2.89 (1.52-5.54) | 0.001 |

BMI, body mass index; FEV_1_, forced expiratory volume in one second; %FEV_1_, ratio of predicted FEV_1_; LAA%, ratio of low attenuation area; WA%, ratio of airway wall area; CRP, C-reactive protein; SAA, serum amyloid A; NLR, Neutrophil-to-Lymphocyte ratio

**Table S3** Predictors of moderate or severe exacerbation by multivariate logistic regression analysis.

|  | Odds ratio (95%CI) | p-value |
| --- | --- | --- |
| Age | 1.04 (1.00-1.08) | 0.079 |
| Smoking Index, pack-years | 1.00 (1.00-1.00) | 0.060 |
| % FEV_1_ < 50% | 2.69 (1.37-5.33) | 0.004 |
| NLR ≥2.7 | 2.22 (1.10-4.46) | 0.025 |

FEV_1_, forced expiratory volume in one second; % FEV_1_, ratio of predicted FEV_1_; NLR, Neutrophil-to-Lymphocyte ratio
